# Supplementary material for: The economic, agricultural, and food security repercussions of a wild pollinator collapse in Europe
Source: Nat Commun. 2025 Nov 10;16:9892. doi: 10.1038/s41467-025-65414-7 (PMC12603210; doi:10.1038/s41467-025-65414-7)
Supplement: Supplementary file 1 — Supplementary Information [file 41467_2025_65414_MOESM1_ESM.pdf]

Overview of Supplementary Materials related to the article "*The Economic, Agricultural, and Food Security Repercussions of a Wild Pollinator Collapse in Europe*" by Feuerbacher et al.

Supplementary Table 1: CAPRI region specific productivity shocks following a wild pollinator collapse in Europe. 2

Supplementary Table 2: Average endogenous yield changes for crop products in Europe following the mean wild pollinator productivity shock 6

Supplementary Figure 1: Global relative change in producer surplus. Source: Authors' analysis based on model results. 7

Supplementary Table 3: Welfare results by component following a wild pollinator collapse in Europe 8

Supplementary Figure 2: Trade balance for selected aggregate CAPRI commodities in the base (reference scenario) and in the wild pollinator collapse in Europe scenario 9

Supplementary Figure 3: Global relative changes in selected food security indicators following a collapse of wild pollinators in Europe. 9

Supplementary Figure 4 - Association between consumer surplus changes and voting behavior on biodiversity-friendly policy proposals. 11

Supplementary Methods 12

1. Sensitivity Analysis of main model results 12

2. Calculation of managed pollinators replacement costs 15

3. Extrapolation of trends in hoverfly, flying insects or terrestrial insect populations in Europe 16

Supplementary Table 1: CAPRI region specific productivity shocks following a wild pollinator collapse in Europe.

Country/Region	CAPRI Commodity	Main scenario - Siopa et al. (2024)			Alternative scenario - Klein et al. (2007)		
		Mean	95% Confidence Interval		Mean	95% Confidence Interval	
			Lower bound	Upper bound		Lower bound	Upper bound
Albania	Apples, pears and peaches	29.73	8.72	37.74	27.90	19.57	36.23
Albania	Citrus fruits	10.08	7.83	12.30	0.78	0.17	1.38
Albania	Other fruits	30.18	21.54	36.54	22.23	15.94	28.48
Albania	Other vegetables	31.04	14.32	51.07	7.90	4.37	11.42
Albania	Pulses	21.28	9.30	31.21	3.55	0.82	6.29
Albania	Soya	9.58	2.11	16.60	12.60	6.73	18.47
Albania	Sunflower	7.92	2.62	12.31	3.67	1.96	5.38
Albania	Tomatoes	13.61	2.99	23.34	2.52	0.56	4.48
Austria	Apples, pears and peaches	31.25	7.97	39.03	27.90	19.57	36.23
Austria	Other fruits	28.60	20.47	32.60	24.40	16.68	32.12
Austria	Other vegetables	41.87	38.81	44.13	39.81	37.20	42.09
Austria	Pulses	20.56	13.92	24.55	4.41	2.00	6.83
Austria	Rapeseed	16.10	4.07	36.07	14.91	7.96	21.86
Austria	Soya	9.58	2.11	16.60	12.60	6.73	18.47
Austria	Sunflower	7.92	2.62	12.31	3.67	1.96	5.38
Austria	Tomatoes	13.61	2.99	23.34	2.52	0.56	4.48
Bosnia and Herzegovina	Apples, pears and peaches	30.00	7.84	38.11	27.90	19.57	36.23
Bosnia and Herzegovina	Citrus fruits	3.09	1.52	4.53	0.78	0.17	1.38
Bosnia and Herzegovina	Other cereals	11.27	11.27	11.27	14.95	10.49	19.42
Bosnia and Herzegovina	Other fruits	28.57	22.26	32.05	26.41	18.53	34.28
Bosnia and Herzegovina	Other vegetables	22.91	11.17	36.38	10.95	6.88	15.02
Bosnia and Herzegovina	Pulses	11.79	5.93	16.88	1.80	0.40	3.20
Bosnia and Herzegovina	Rapeseed	16.10	4.07	36.07	14.91	7.96	21.86
Bosnia and Herzegovina	Soya	9.58	2.11	16.60	12.60	6.73	18.47
Bosnia and Herzegovina	Sunflower	7.92	2.62	12.31	3.67	1.96	5.38
Bosnia and Herzegovina	Tomatoes	13.61	2.99	23.34	2.52	0.56	4.48
Belarus	Apples, pears and peaches	31.39	7.14	39.13	27.90	19.57	36.23
Belarus	Other cereals	7.02	7.02	7.02	9.31	6.53	12.10
Belarus	Other fruits	21.89	16.01	26.39	14.72	9.71	19.73
Belarus	Other vegetables	13.82	8.83	17.97	20.65	14.49	26.82
Belarus	Pulses	14.27	3.15	24.72	3.75	0.84	6.67
Belarus	Rapeseed	16.10	4.07	36.07	14.91	7.96	21.86
Belarus	Sunflower	7.92	2.62	12.31	3.67	1.96	5.38
Belarus	Tomatoes	13.61	2.99	23.34	2.52	0.56	4.48
Bulgaria	Apples, pears and peaches	24.43	6.78	34.14	27.90	19.57	36.23
Bulgaria	Other fruits	29.65	24.01	33.20	29.05	22.10	35.93
Bulgaria	Other vegetables	8.39	5.11	11.83	7.25	5.16	9.34
Bulgaria	Pulses	8.86	8.34	9.33	0.14	0.03	0.25
Bulgaria	Rapeseed	16.10	4.07	36.07	14.91	7.96	21.86
Bulgaria	Soya	9.58	2.11	16.60	12.60	6.73	18.47
Bulgaria	Sunflower	7.92	2.62	12.31	3.67	1.96	5.38
Bulgaria	Tomatoes	13.61	2.99	23.34	2.52	0.56	4.48
Belgium and Luxembourg	Apples, pears and peaches	31.94	11.11	39.40	27.90	19.57	36.23
Belgium and Luxembourg	Other fruits	25.60	19.70	32.31	15.50	9.31	21.70
Belgium and Luxembourg	Other vegetables	9.34	8.19	10.49	7.72	7.14	8.23
Belgium and Luxembourg	Pulses	7.76	5.05	9.33	0.54	0.13	0.94
Belgium and Luxembourg	Rapeseed	16.10	4.07	36.07	14.91	7.96	21.86
Belgium and Luxembourg	Tomatoes	13.61	2.99	23.34	2.52	0.56	4.48
Switzerland	Apples, pears and peaches	31.36	7.47	39.09	27.90	19.57	36.23
Switzerland	Other fruits	28.56	19.28	33.83	22.75	15.30	30.20
Switzerland	Other vegetables	4.73	4.07	5.25	5.02	4.34	5.68
Switzerland	Pulses	3.99	2.59	4.81	0.54	0.21	0.87
Switzerland	Rapeseed	16.10	4.07	36.07	14.91	7.96	21.86
Switzerland	Soya	9.58	2.11	16.60	12.60	6.73	18.47
Switzerland	Sunflower	7.92	2.62	12.31	3.67	1.96	5.38
Switzerland	Tomatoes	13.61	2.99	23.34	2.52	0.56	4.48
Serbia	Apples, pears and peaches	29.50	9.44	37.47	27.90	19.57	36.23
Serbia	Other fruits	26.79	9.23	31.52	30.86	21.69	40.03
Serbia	Other vegetables	34.42	13.65	59.58	4.03	1.13	6.94
Serbia	Pulses	22.78	14.76	27.46	1.50	0.34	2.67
Serbia	Rapeseed	16.10	4.07	36.07	14.91	7.96	21.86
Serbia	Soya	9.58	2.11	16.60	12.60	6.73	18.47
Serbia	Sunflower	7.92	2.62	12.31	3.67	1.96	5.38
Serbia	Tomatoes	13.61	2.99	23.34	2.52	0.56	4.48
Cyprus	Apples, pears and peaches	26.16	7.19	35.36	27.90	19.57	36.23
Cyprus	Citrus fruits	10.12	7.48	12.47	0.78	0.17	1.38
Cyprus	Other fruits	24.53	21.41	26.70	21.01	17.22	24.70
Cyprus	Other vegetables	9.29	7.47	11.06	8.89	6.66	11.10

Country/Region	CAPRI Commodity	Main scenario - Siopa et al. (2024)			Alternative scenario - Klein et al. (2007)		
		Mean	95% Confidence Interval		Mean	95% Confidence Interval	
			Lower bound	Upper bound		Lower bound	Upper bound
Cyprus	Pulses	42.39	27.59	51.32	3.21	0.84	5.58
Cyprus	Tomatoes	13.61	2.99	23.34	2.52	0.56	4.48
Czech Republic	Apples, pears and peaches	31.19	6.76	38.99	27.90	19.57	36.23
Czech Republic	Other cereals	10.32	10.32	10.32	13.69	9.60	17.78
Czech Republic	Other fruits	13.95	12.71	14.97	10.69	6.62	14.76
Czech Republic	Other vegetables	9.20	4.93	13.63	8.39	5.70	11.09
Czech Republic	Pulses	2.76	1.98	3.22	0.35	0.15	0.56
Czech Republic	Rapeseed	16.10	4.07	36.07	14.91	7.96	21.86
Czech Republic	Soya	9.58	2.11	16.60	12.60	6.73	18.47
Czech Republic	Sunflower	7.92	2.62	12.31	3.67	1.96	5.38
Czech Republic	Tomatoes	13.61	2.99	23.34	2.52	0.56	4.48
Germany	Apples, pears and peaches	31.32	6.73	39.07	27.90	19.57	36.23
Germany	Other fruits	20.81	15.92	26.11	13.77	8.52	19.03
Germany	Other vegetables	7.06	5.35	8.74	6.72	5.53	7.88
Germany	Pulses	1.98	1.27	2.42	1.08	0.54	1.61
Germany	Rapeseed	16.10	4.07	36.07	14.91	7.96	21.86
Germany	Soya	9.58	2.11	16.60	12.60	6.73	18.47
Germany	Sunflower	7.92	2.62	12.31	3.67	1.96	5.38
Germany	Tomatoes	13.61	2.99	23.34	2.52	0.56	4.48
Denmark	Apples, pears and peaches	31.45	7.78	39.17	27.90	19.57	36.23
Denmark	Other fruits	27.82	21.60	35.06	16.23	9.81	22.65
Denmark	Other vegetables	5.25	3.35	6.82	7.84	5.50	10.18
Denmark	Pulses	1.00	1.00	1.00	1.08	0.54	1.61
Denmark	Rapeseed	16.10	4.07	36.07	14.91	7.96	21.86
Denmark	Tomatoes	13.61	2.99	23.34	2.52	0.56	4.48
Estonia	Apples, pears and peaches	31.33	6.49	39.09	27.90	19.57	36.23
Estonia	Other cereals	10.95	10.95	10.96	14.53	10.19	18.87
Estonia	Other fruits	25.36	20.84	30.71	16.62	10.13	23.12
Estonia	Other vegetables	8.57	5.48	11.14	12.80	8.98	16.63
Estonia	Pulses	2.71	0.60	4.69	0.71	0.16	1.27
Estonia	Rapeseed	16.10	4.07	36.07	14.91	7.96	21.86
Estonia	Tomatoes	13.61	2.99	23.34	2.52	0.56	4.48
Greece	Apples, pears and peaches	24.77	7.70	34.38	27.90	19.57	36.23
Greece	Citrus fruits	5.94	4.24	7.55	0.78	0.17	1.38
Greece	Other fruits	25.17	18.87	28.82	20.53	15.78	25.22
Greece	Other vegetables	14.66	9.62	20.30	9.96	6.77	13.15
Greece	Pulses	12.03	7.77	14.93	1.02	0.25	1.80
Greece	Rapeseed	16.10	4.07	36.07	14.91	7.96	21.86
Greece	Soya	9.58	2.11	16.60	12.60	6.73	18.47
Greece	Sunflower	7.92	2.62	12.31	3.67	1.96	5.38
Greece	Tomatoes	13.61	2.99	23.34	2.52	0.56	4.48
Spain	Apples, pears and peaches	21.19	7.19	31.83	27.90	19.57	36.23
Spain	Citrus fruits	7.75	6.08	9.34	0.78	0.17	1.38
Spain	Other fruits	30.69	22.53	35.45	25.56	19.65	31.39
Spain	Other vegetables	25.08	18.18	32.95	15.76	12.42	19.03
Spain	Pulses	33.07	22.03	39.80	2.50	0.68	4.33
Spain	Rapeseed	16.10	4.07	36.07	14.91	7.96	21.86
Spain	Soya	9.58	2.11	16.60	12.60	6.73	18.47
Spain	Sunflower	7.92	2.62	12.31	3.67	1.96	5.38
Spain	Tomatoes	13.61	2.99	23.34	2.52	0.56	4.48
Finland	Apples, pears and peaches	31.33	6.49	39.09	27.90	19.57	36.23
Finland	Other fruits	25.45	16.92	31.66	18.52	11.69	25.36
Finland	Other vegetables	5.51	3.92	6.88	6.89	5.19	8.58
Finland	Pulses	0.36	0.24	0.44	0.02	0.01	0.04
Finland	Rapeseed	16.10	4.07	36.07	14.91	7.96	21.86
Finland	Tomatoes	13.61	2.99	23.34	2.52	0.56	4.48
France	Apples, pears and peaches	28.82	6.93	37.29	27.90	19.57	36.23
France	Citrus fruits	10.32	8.72	11.88	0.78	0.17	1.38
France	Other cereals	10.27	10.27	10.27	13.62	9.55	17.69
France	Other fruits	35.52	32.24	37.06	31.95	24.48	39.35
France	Other vegetables	8.51	8.02	8.82	8.25	7.68	8.75
France	Pulses	0.26	0.12	0.39	0.44	0.22	0.65
France	Rapeseed	16.10	4.07	36.07	14.91	7.96	21.86
France	Soya	9.58	2.11	16.60	12.60	6.73	18.47
France	Sunflower	7.92	2.62	12.31	3.67	1.96	5.38
France	Tomatoes	13.61	2.99	23.34	2.52	0.56	4.48
Croatia	Apples, pears and peaches	28.67	6.98	37.16	27.90	19.57	36.23
Croatia	Citrus fruits	11.49	8.57	14.42	0.78	0.17	1.38
Croatia	Other cereals	6.43	6.43	6.43	8.53	5.98	11.08
Croatia	Other fruits	22.04	17.89	25.14	23.52	16.96	30.07
Croatia	Other vegetables	9.10	4.54	14.10	6.38	4.22	8.55
Croatia	Pulses	30.63	18.24	38.90	2.76	0.62	4.91
Croatia	Rapeseed	16.10	4.07	36.07	14.91	7.96	21.86
Croatia	Soya	9.58	2.11	16.60	12.60	6.73	18.47

Country/Region	CAPRI Commodity	Main scenario - Siopa et al. (2024)			Alternative scenario - Klein et al. (2007)		
		Mean	95% Confidence Interval		Mean	95% Confidence Interval	
			Lower bound	Upper bound		Lower bound	Upper bound
Croatia	Sunflower	7.92	2.62	12.31	3.67	1.96	5.38
Croatia	Tomatoes	13.61	2.99	23.34	2.52	0.56	4.48
Hungary	Apples, pears and peaches	30.30	7.15	38.31	27.90	19.57	36.23
Hungary	Other cereals	9.40	9.40	9.40	12.47	8.75	16.19
Hungary	Other fruits	31.45	24.12	35.85	27.79	21.40	34.11
Hungary	Other vegetables	20.50	9.71	33.17	7.37	4.73	10.00
Hungary	Pulses	4.25	2.68	5.23	0.35	0.09	0.61
Hungary	Rapeseed	16.10	4.07	36.07	14.91	7.96	21.86
Hungary	Soya	9.58	2.11	16.60	12.60	6.73	18.47
Hungary	Sunflower	7.92	2.62	12.31	3.67	1.96	5.38
Hungary	Tomatoes	13.61	2.99	23.34	2.52	0.56	4.48
Irland	Apples, pears and peaches	31.33	6.49	39.09	27.90	19.57	36.23
Irland	Other fruits	25.67	20.89	32.94	13.01	7.23	18.79
Irland	Other vegetables	3.80	2.29	5.20	4.88	3.39	6.36
Irland	Pulses	11.87	7.54	14.70	9.16	4.69	13.64
Irland	Rapeseed	16.10	4.07	36.07	14.91	7.96	21.86
Irland	Tomatoes	13.61	2.99	23.34	2.52	0.56	4.48
Italy	Apples, pears and peaches	19.06	7.12	30.32	27.90	19.57	36.23
Italy	Citrus fruits	9.47	7.04	11.86	0.78	0.17	1.38
Italy	Other fruits	36.40	30.93	40.23	28.56	21.43	35.64
Italy	Other vegetables	12.15	10.63	13.66	10.24	9.47	10.92
Italy	Pulses	30.24	19.71	36.35	2.23	0.59	3.87
Italy	Rapeseed	16.10	4.07	36.07	14.91	7.96	21.86
Italy	Soya	9.58	2.11	16.60	12.60	6.73	18.47
Italy	Sunflower	7.92	2.62	12.31	3.67	1.96	5.38
Italy	Tomatoes	13.61	2.99	23.34	2.52	0.56	4.48
Lithuania	Apples, pears and peaches	31.48	7.09	39.14	27.90	19.57	36.23
Lithuania	Other cereals	9.58	9.58	9.58	12.71	8.92	16.51
Lithuania	Other fruits	23.55	20.12	26.44	29.61	20.59	38.63
Lithuania	Other vegetables	27.26	23.32	30.35	30.03	25.05	34.87
Lithuania	Pulses	7.30	3.33	10.73	1.21	0.27	2.15
Lithuania	Rapeseed	16.10	4.07	36.07	14.91	7.96	21.86
Lithuania	Tomatoes	13.61	2.99	23.34	2.52	0.56	4.48
Latvia	Apples, pears and peaches	32.17	9.46	39.36	27.90	19.57	36.23
Latvia	Other cereals	9.75	9.75	9.76	12.94	9.08	16.80
Latvia	Other fruits	25.09	21.27	29.66	24.94	16.92	32.96
Latvia	Other vegetables	12.22	9.08	14.75	16.23	12.34	20.08
Latvia	Pulses	11.37	2.52	19.69	2.99	0.67	5.31
Latvia	Rapeseed	16.10	4.07	36.07	14.91	7.96	21.86
Latvia	Tomatoes	13.61	2.99	23.34	2.52	0.56	4.48
Macedonia	Apples, pears and peaches	29.45	9.89	37.39	27.90	19.57	36.23
Macedonia	Other fruits	22.72	17.92	25.54	19.64	15.10	24.12
Macedonia	Other vegetables	15.37	7.16	24.80	7.29	4.55	10.03
Macedonia	Pulses	40.09	25.46	48.97	2.89	0.65	5.13
Macedonia	Rapeseed	16.10	4.07	36.07	14.91	7.96	21.86
Macedonia	Soya	9.58	2.11	16.60	12.60	6.73	18.47
Macedonia	Sunflower	7.92	2.62	12.31	3.67	1.96	5.38
Macedonia	Tomatoes	13.61	2.99	23.34	2.52	0.56	4.48
Montenegro	Apples, pears and peaches	25.42	8.24	34.86	27.90	19.57	36.23
Montenegro	Citrus fruits	4.42	2.61	6.11	0.78	0.17	1.38
Montenegro	Other fruits	32.35	27.61	34.66	30.00	21.60	38.37
Montenegro	Other vegetables	8.62	3.39	14.96	0.90	0.20	1.60
Montenegro	Pulses	24.26	15.59	29.40	1.64	0.37	2.92
Montenegro	Tomatoes	13.61	2.99	23.34	2.52	0.56	4.48
Malta	Apples, pears and peaches	16.80	6.57	28.71	27.90	19.57	36.23
Malta	Citrus fruits	6.65	5.37	7.84	0.78	0.17	1.38
Malta	Other fruits	31.16	27.69	35.08	29.41	25.88	32.72
Malta	Other vegetables	9.69	8.46	10.86	8.83	7.18	10.43
Malta	Pulses	7.65	2.36	12.68	4.74	2.08	7.40
Malta	Tomatoes	13.61	2.99	23.34	2.52	0.56	4.48
The Netherlands	Apples, pears and peaches	31.59	9.28	39.26	27.90	19.57	36.23
The Netherlands	Other fruits	27.72	19.31	32.74	23.41	15.71	31.10
The Netherlands	Other vegetables	24.40	16.99	32.69	16.40	13.25	19.46
The Netherlands	Pulses	34.30	22.33	41.20	2.23	0.51	3.96
The Netherlands	Rapeseed	16.10	4.07	36.07	14.91	7.96	21.86
The Netherlands	Tomatoes	13.61	2.99	23.34	2.52	0.56	4.48
Norway	Apples, pears and peaches	31.34	6.63	39.10	27.90	19.57	36.23
Norway	Other fruits	27.37	18.90	33.74	19.57	12.50	26.64
Norway	Other vegetables	4.55	2.91	5.92	6.80	4.77	8.84
Norway	Pulses	11.55	7.53	13.86	0.74	0.17	1.32
Norway	Rapeseed	16.10	4.07	36.07	14.91	7.96	21.86
Norway	Tomatoes	13.61	2.99	23.34	2.52	0.56	4.48
Poland	Apples, pears and peaches	31.30	6.74	39.06	27.90	19.57	36.23
Poland	Other cereals	0.38	0.38	0.38	0.51	0.36	0.66

Country/Region	CAPRI Commodity	Main scenario - Siopa et al. (2024)			Alternative scenario - Klein et al. (2007)		
		Mean	95% Confidence Interval		Mean	95% Confidence Interval	
			Lower bound	Upper bound		Lower bound	Upper bound
Poland	Other fruits	23.03	18.62	26.50	24.85	17.10	32.61
Poland	Other vegetables	16.36	15.24	16.99	16.81	15.39	18.10
Poland	Pulses	4.22	2.94	5.14	1.27	0.60	1.94
Poland	Rapeseed	16.10	4.07	36.07	14.91	7.96	21.86
Poland	Soya	9.58	2.11	16.60	12.60	6.73	18.47
Poland	Sunflower	7.92	2.62	12.31	3.67	1.96	5.38
Poland	Tomatoes	13.61	2.99	23.34	2.52	0.56	4.48
Portugal	Apples, pears and peaches	30.30	8.72	38.29	27.90	19.57	36.23
Portugal	Citrus fruits	4.89	3.20	6.48	0.78	0.17	1.38
Portugal	Other fruits	28.80	18.49	33.78	28.03	21.41	34.58
Portugal	Other vegetables	1.09	0.85	1.35	0.77	0.61	0.94
Portugal	Pulses	13.15	7.81	16.96	1.57	0.51	2.63
Portugal	Sunflower	7.92	2.62	12.31	3.67	1.96	5.38
Portugal	Tomatoes	13.61	2.99	23.34	2.52	0.56	4.48
Rest of Europe	Other vegetables	19.81	12.66	25.75	29.60	20.76	38.44
Rest of Europe	Tomatoes	13.61	2.99	23.34	2.52	0.56	4.48
Romania	Apples, pears and peaches	31.04	8.76	38.70	27.90	19.57	36.23
Romania	Other fruits	30.63	27.03	33.09	28.62	21.96	35.20
Romania	Other vegetables	13.85	8.91	19.66	7.10	4.71	9.48
Romania	Pulses	8.44	5.18	10.57	0.70	0.16	1.25
Romania	Rapeseed	16.10	4.07	36.07	14.91	7.96	21.86
Romania	Soya	9.58	2.11	16.60	12.60	6.73	18.47
Romania	Sunflower	7.92	2.62	12.31	3.67	1.96	5.38
Romania	Tomatoes	13.61	2.99	23.34	2.52	0.56	4.48
Russia	Apples, pears and peaches	30.81	6.61	38.71	27.90	19.57	36.23
Russia	Citrus fruits	2.95	1.37	4.38	0.78	0.17	1.38
Russia	Other cereals	10.55	10.55	10.55	14.00	9.82	18.18
Russia	Other fruits	26.73	20.57	30.36	20.05	12.95	27.15
Russia	Other vegetables	50.96	48.07	52.44	51.46	47.75	54.75
Russia	Pulses	2.27	2.20	2.33	0.05	0.02	0.08
Russia	Rapeseed	16.10	4.07	36.07	14.91	7.96	21.86
Russia	Soya	9.58	2.11	16.60	12.60	6.73	18.47
Russia	Sunflower	7.92	2.62	12.31	3.67	1.96	5.38
Russia	Tomatoes	13.61	2.99	23.34	2.52	0.56	4.48
Sweden	Apples, pears and peaches	31.46	7.92	39.18	27.90	19.57	36.23
Sweden	Other fruits	25.84	19.35	31.49	17.26	10.64	23.88
Sweden	Other vegetables	2.06	1.31	2.67	3.07	2.16	3.99
Sweden	Pulses	0.31	0.17	0.46	1.56	0.84	2.29
Sweden	Rapeseed	16.10	4.07	36.07	14.91	7.96	21.86
Sweden	Tomatoes	13.61	2.99	23.34	2.52	0.56	4.48
Slovenia	Apples, pears and peaches	30.75	6.79	38.67	27.90	19.57	36.23
Slovenia	Other cereals	10.13	10.13	10.13	13.44	9.42	17.45
Slovenia	Other fruits	16.95	13.17	20.51	14.20	9.59	18.82
Slovenia	Other vegetables	6.49	2.61	11.17	0.98	0.38	1.59
Slovenia	Pulses	13.73	8.18	17.43	1.24	0.28	2.21
Slovenia	Rapeseed	16.10	4.07	36.07	14.91	7.96	21.86
Slovenia	Soya	9.58	2.11	16.60	12.60	6.73	18.47
Slovenia	Sunflower	7.92	2.62	12.31	3.67	1.96	5.38
Slovenia	Tomatoes	13.61	2.99	23.34	2.52	0.56	4.48
Slovak Republic	Apples, pears and peaches	30.35	6.63	38.38	27.90	19.57	36.23
Slovak Republic	Other cereals	10.00	10.00	10.00	13.26	9.30	17.22
Slovak Republic	Other fruits	26.50	21.79	29.27	25.32	20.30	30.25
Slovak Republic	Other vegetables	41.34	37.79	44.21	38.66	36.05	40.94
Slovak Republic	Pulses	0.33	0.24	0.41	0.19	0.09	0.28
Slovak Republic	Rapeseed	16.10	4.07	36.07	14.91	7.96	21.86
Slovak Republic	Soya	9.58	2.11	16.60	12.60	6.73	18.47
Slovak Republic	Sunflower	7.92	2.62	12.31	3.67	1.96	5.38
Slovak Republic	Tomatoes	13.61	2.99	23.34	2.52	0.56	4.48
Ukraine	Apples, pears and peaches	31.14	7.53	38.92	27.90	19.57	36.23
Ukraine	Other cereals	9.20	9.20	9.20	12.20	8.56	15.84
Ukraine	Other fruits	16.94	13.97	18.06	15.47	13.41	17.43
Ukraine	Other vegetables	61.94	57.43	65.42	57.54	54.18	60.39
Ukraine	Pulses	6.97	2.77	10.59	1.41	0.36	2.47
Ukraine	Rapeseed	16.10	4.07	36.07	14.91	7.96	21.86
Ukraine	Soya	9.58	2.11	16.60	12.60	6.73	18.47
Ukraine	Sunflower	7.92	2.62	12.31	3.67	1.96	5.38
Ukraine	Tomatoes	13.61	2.99	23.34	2.52	0.56	4.48
Simple Mean (all regions and commodities)		*16.58*	*8.04*	*23.67*	*12.24*	*8.10*	*16.37*

Note: Regions outside of Europe are not shocked. Source: Authors' analysis, see methods for more details.

Supplementary Table 2: Average endogenous yield changes for crop products in Europe following the mean wild pollinator productivity shock.

Note: The CAPRI supply model distinguishes within each region between an intensive and extensive type of agricultural production technologies[1]. The technologies are split in a stylized manner such that their weighted average replicate the respective region's means[2]. In addition, the model uses an isoelastic function to capture overall endogenous yield changes subject to changes in output prices. This function is part of the convergence mechanism between the supply and market module of the CAPRI model[3]. In the market model, the model decomposes production changes into changes in land demand and yield, for which region-specific land supply and yield elasticities are used.

CAPRI Commodity	Endogenous yield change (in %)
Crop Products*	0.65
Pollination Dependent Crops*	2.14
Wheat	0.10
Barley	0.11
Maize	0.09
Rice	-0.05
Rye	0.25
Oats	0.22
Other Cereals	0.97
Rapeseed	2.95
Sunflower	2.40
Soya	2.79
Pulses	0.94
Potatos	0.14
Tomatoes	2.95
Other vegetables	5.24
Apple, Pears and Peaches	4.14
Citrus Fruits	-0.04
Other Fruits	0.60
Table Olives	0.04
Olive oil	0.05
Table grapes	0.08
Wine	0.02
Tobacco	0.01
Textile	-0.12
Sugar	-0.05

Source: Authors' analysis based on model results.

*Denotes aggregates, which are weighted averages by land area

Supplementary Figure 1: Global relative change in producer surplus. Source: Authors' analysis based on model results.

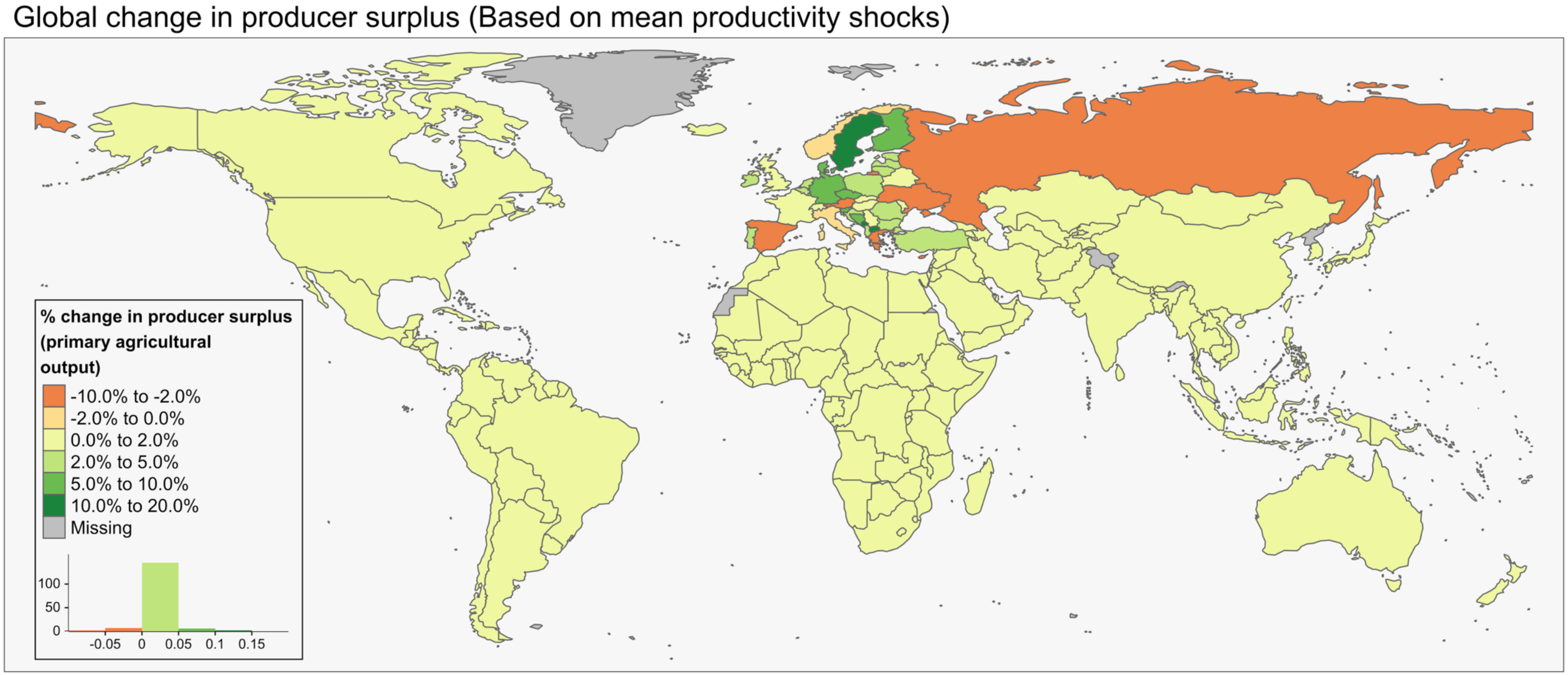

Source: Administrative and territorial boundaries based on © EuroGeographics 2025.

Supplementary Table 3: Welfare results by component following a wild pollinator collapse in Europe

Region	Welfare Indicator	Base value in million EUR	Main scenario based on Siopa et al. (2024) - absolute change in million EUR			Alternative scenario based on Klein et al. (2007) - absolute change in million EUR			Main scenario based on Siopa et al. (2024) - relative change compared to base in %		
			Mean	Lower	Upper	Mean	Lower	Upper	Mean	Lower	Upper
Global	Total Welfare	6,759,725	-34,425	-23,225	-44,026	-27,307	-21,407	-33,295	-0.5%	-0.3%	-0.7%
	Producer Surplus - Farm	1,593,298	4,585	782	9,500	2,682	1,417	4,023	0.3%	0.0%	0.6%
	Consumer Surplus	4,938,384	-36,808	-23,028	-49,846	-27,738	-21,254	-34,602	-0.7%	-0.5%	-1.0%
	Rents Processing	194,937	-3,410	-1,845	-5,224	-3,104	-2,252	-3,761	-1.7%	-0.9%	-2.7%
	Government	33,107	1,208	866	1,544	853	682	1,045	3.6%	2.6%	4.7%
Europe	Total Welfare	1,096,170	-23,845	-16,184	-30,632	-18,811	-14,868	-22,795	-2.2%	-1.5%	-2.8%
	Producer Surplus - Farm	175,345	-526	-2,012	1,551	-1,136	-1,219	-1,056	-0.3%	-1.1%	0.9%
	Consumer Surplus	896,554	-21,938	-13,613	-29,872	-16,146	-12,569	-19,984	-2.4%	-1.5%	-3.3%
	Rents Processing	28,664	-2,598	-1,448	-3,827	-2,432	-1,819	-2,839	-9.1%	-5.1%	-13.4%
	Government	-4,393	1,218	889	1,516	903	738	1,085	-27.7%	-20.2%	-34.5%
EU	Total Welfare	752,507	-12,419	-7,678	-16,835	-8,766	-6,419	-11,082	-1.7%	-1.0%	-2.2%
	Producer Surplus - Farm	117,537	330	-805	1,724	-64	-124	54	0.3%	-0.7%	1.5%
	Consumer Surplus	623,792	-12,592	-7,059	-17,942	-8,393	-6,139	-10,892	-2.0%	-1.1%	-2.9%
	Rents Processing	20,634	-809	-298	-1,433	-750	-506	-789	-3.9%	-1.4%	-6.9%
	Government	-9,457	654	484	817	441	351	546	-6.9%	-5.1%	-8.6%
Extra-Europe	Total Welfare	5,663,555	-10,580	-7,041	-13,393	-8,496	-6,539	-10,500	-0.2%	-0.1%	-0.2%
	Producer Surplus - Farm	1,417,953	5,112	2,794	7,949	3,818	2,636	5,080	0.4%	0.2%	0.6%
	Consumer Surplus	4,041,829	-14,870	-9,414	-19,974	-11,592	-8,685	-14,618	-0.4%	-0.2%	-0.5%
	Rents Processing	166,273	-812	-397	-1,396	-672	-433	-921	-0.5%	-0.2%	-0.8%
	Government	37,500	-10	-23	28	-50	-56	-40	0.0%	-0.1%	0.1%

Note: The main scenario relies on the dependence ratios reported by Siopa et al.[4], while the alternative scenario uses the dependence ratios from Klein et al.[5]

Supplementary Figure 2: Trade balance for selected aggregate CAPRI commodities in the base (reference scenario) and in the wild pollinator collapse in Europe scenario (Mean productivity shock)

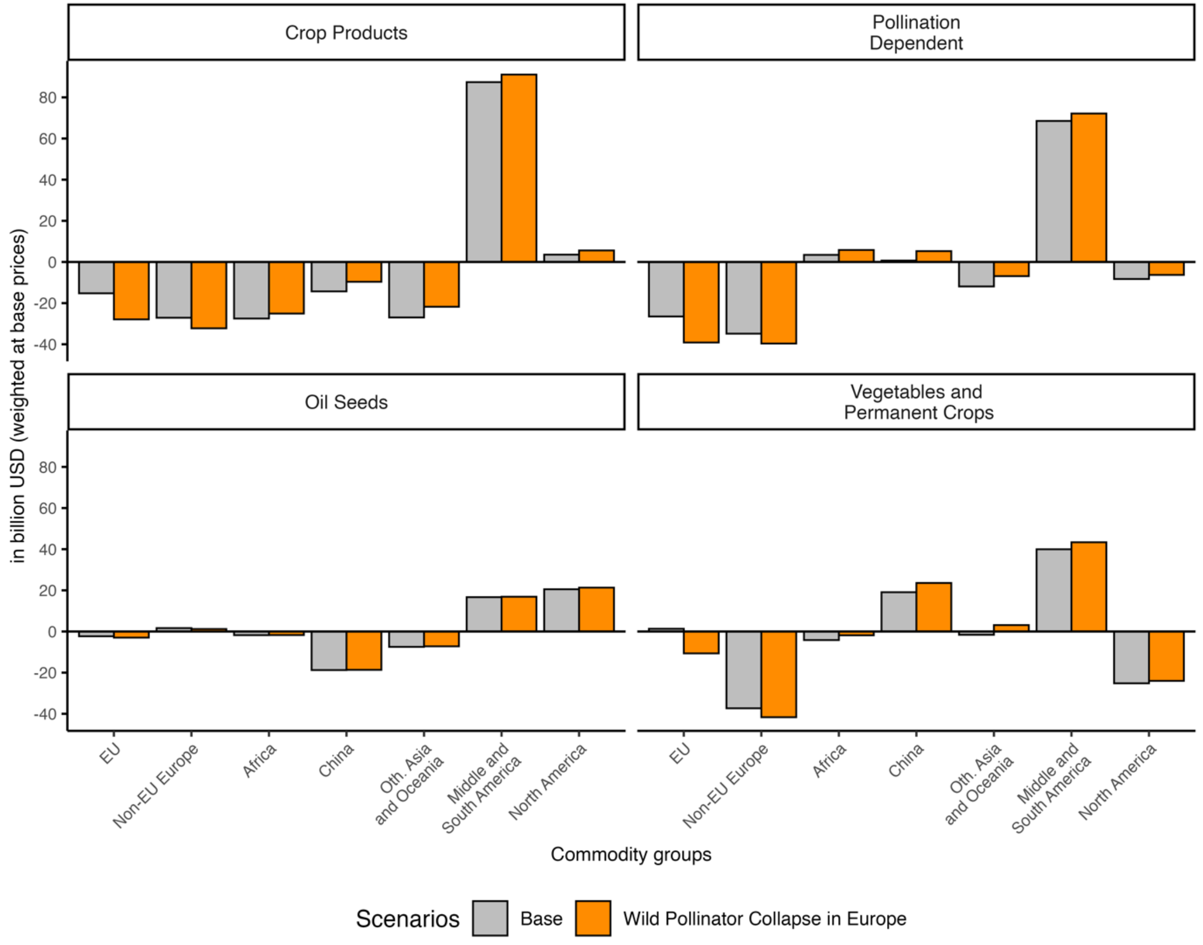

Source: Authors' analysis based on model results.

Supplementary Figure 3: Global relative changes in selected food security indicators following a collapse of wild pollinators in Europe.

A) Global relative changes in Vitamin-A availability in international units per capita and day

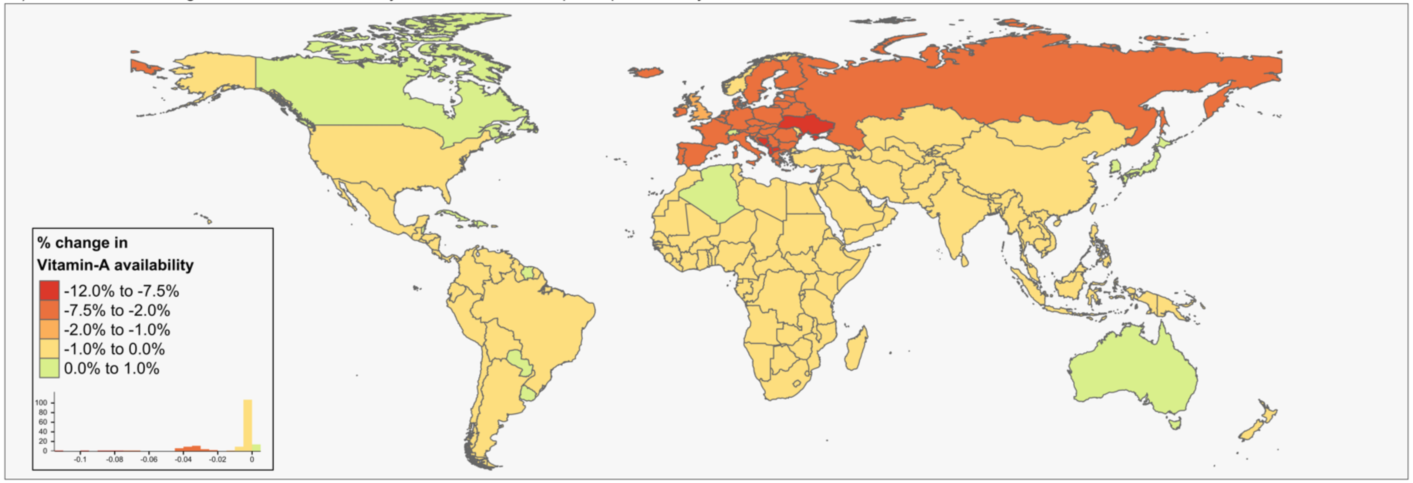

B) Global relative changes in food intake (kg per capita and day after losses)

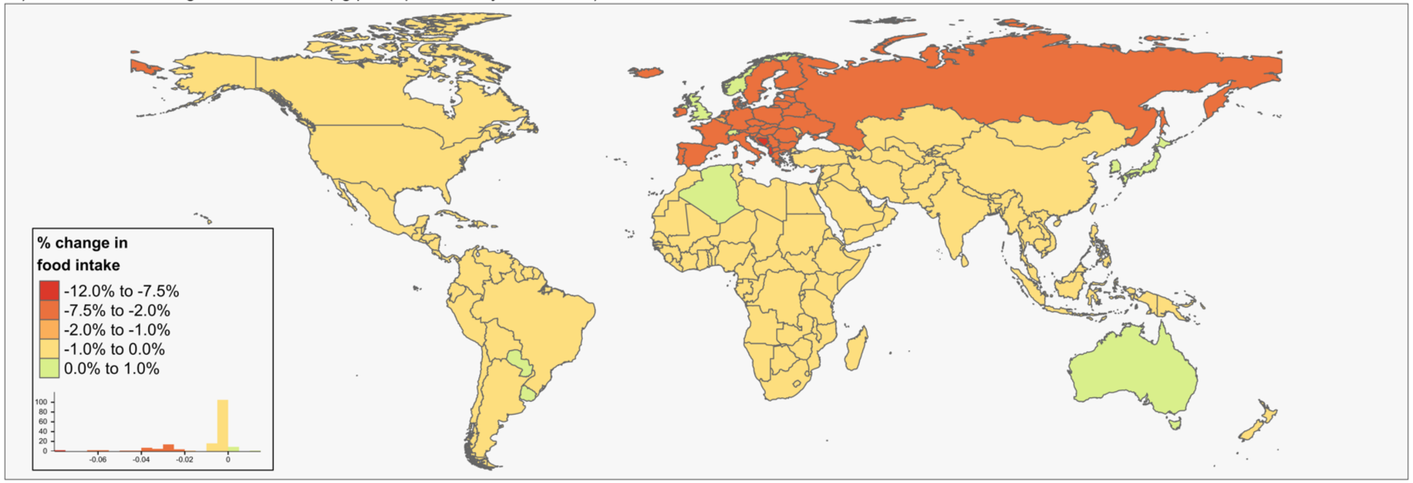

Source: Authors' analysis based on model results. Administrative and territorial boundaries based on © EuroGeographics 2025.

Supplementary Figure 4: Association between consumer surplus changes and voting behavior on biodiversity-friendly policy proposals.

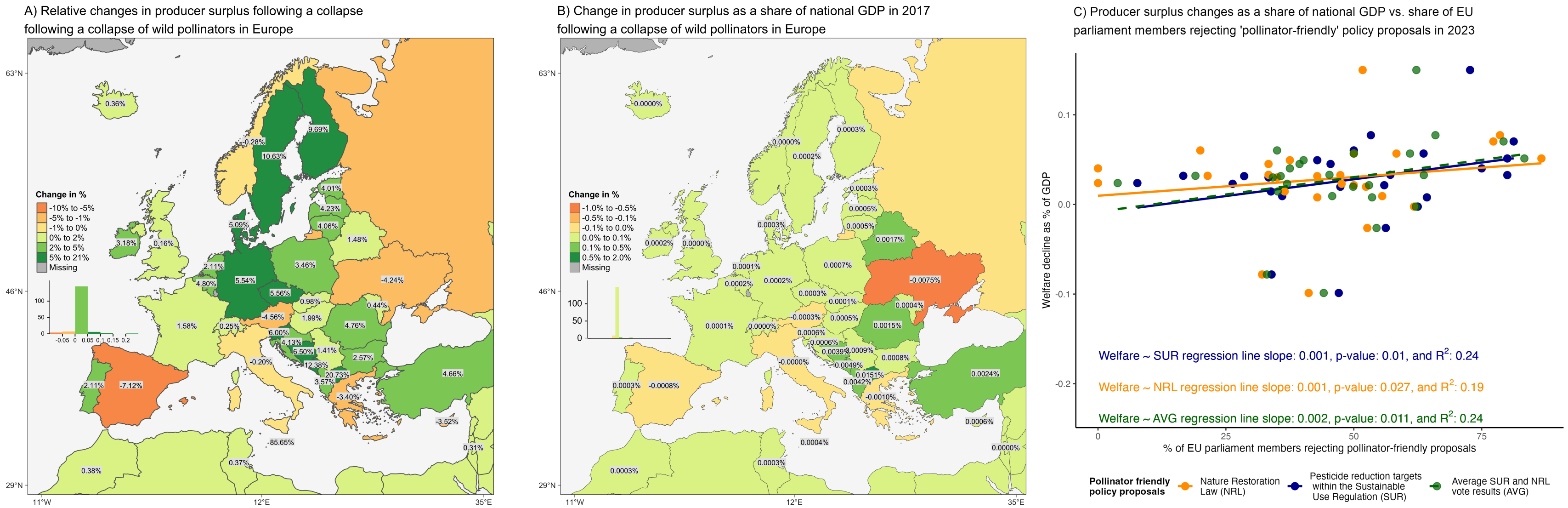

Panel A) Relative changes in producer surplus following a wild pollinator collapse. B) Changes in consumer welfare as a share in national gross domestic product. C) Scatter plot and the population-weighted statistical relationship between consumer surplus changes and shares of EU parliament members rejecting the NRL or SUR based on 26 member states (n=26). Luxembourg is included within Belgium in the CAPRI model. Source: Authors' own analysis based on model results and the European Parliament [6,7]. Administrative and territorial boundaries based on © EuroGeographics 2025.

Supplementary Methods

1. Sensitivity analysis of main model results

Following Jansson et al. (2020) we conduct a systematic sensitivity analysis to test the robustness of the model results following a simulated collapse of wild pollinators. We identified the four parameters (Supplementary Table 4) that we consider most crucial for the main model results. Each of the parameters is varied from its "mean" value to a low or high level.

Supplementary Table 4 – Description of parameters varied within the sensitivity analysis

Parameter	Description	Variation
Supply Elasticity	The supply elasticities of agricultural production within Europe are determined by the region specific slope of the marginal cost functions within the model's supply module. Following Jansson et al.[8] we vary the coefficient of the quadratic term of the cost functions of the Positive Mathematical Programming models within the supply module.	The supply elasticities are adjusted by modifying the slope of the marginal cost function by a relative change of ±50%. The "high" elasticity level represents a more elastic supply scenario, where the slope is decreased by 50%. Conversely, the "low" elasticity level represents a more inelastic supply scenario, where the slope is increased by 50%.
Armington trade elasticities	The market module of the CAPRI models uses CES (constant elasticity of substitution) elasticities to govern the degree of substitutability between imports and domestic goods.	The standard CES elasticities as determined in the CAPRI model calibration are varied by a relative change of +/- 50%.
Contribution of wild pollinators	The contribution of wild pollinators within Europe for different crop groups is estimated based on data from Reilly et al.[9] However, for some crops, only limited data points are available. Across all crops, the median contribution of wild pollinators is 49.7%, with an interquartile range of 22.5% to 84.8%, indicating a relative variation of -43.5% and +46.8% around the median.	We apply the interquartile range of all crops (see left column), adjusting the contribution of wild pollinators within Europe by a relative change of -43.5% (low) and +46.8% (high).
Yield dependency on pollination services	The dependence ratios from Siopa et al. [4] indicate the extent to which yields rely on both wild and managed pollinators. A 95% confidence interval for these ratios is estimated using a triangular distribution, based on the reported minimum and maximum values for the dependence ratios.	The "high" yield dependency refers to upper bound of the estimated 95% confidence interval of crop-specific dependence ratios, while the "low" level refers to the lower bound. See Table 1 for the different crop commodities in the CAPRI model that are pollination dependent.

We lack information on possible correlations or covariances between the four parameters across regions and crops, so we assume they are statistically independent. Each parameter is varied uniformly across all crop commodities and regions simultaneously[8]. For example, a scenario with high wild pollinator contribution increases this contribution by 46.8% for all relevant crops and regions at once. This setup yields 81 model variations (3 discrete levels for each of the 4 parameters), allowing for an efficient sensitivity analysis, as each model run takes approximately 40 minutes on our server, totaling around 54 hours of runtime.

A more extensive stochastic analysis, incorporating intermediate levels of parameter variation, typically requires 1000 or more iterations—leading to unmanageable runtimes and large result files. Our approach captures a broad result space efficiently without needing additional levels

of parameter variation. We focus on the variation in results of four key indicators, which are changes in:

- Consumer surplus as a share of GDP
- Total welfare as a share of GDP
- Vegetable and fruit production
- Vitamin A intake

Supplementary Figure 5 and 6 illustrate the results of the sensitivity analysis for the key result indicators.

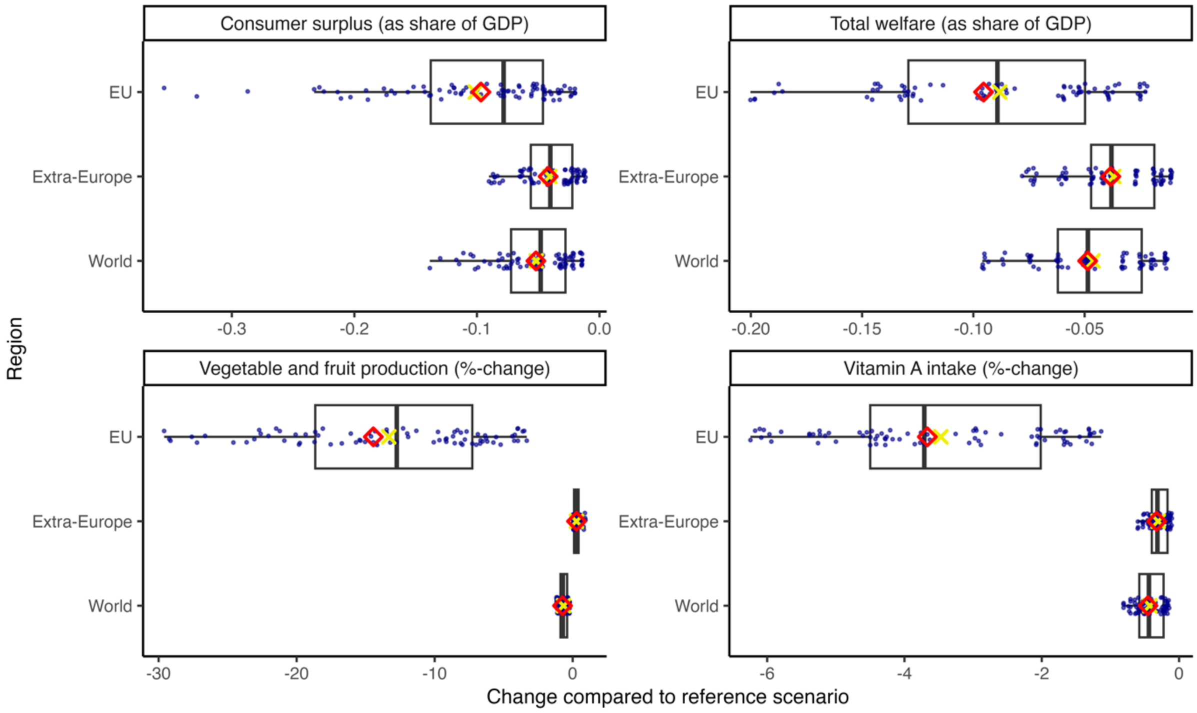

Supplementary Figure 5– Sensitivity analysis of main model results after varying key model parameters. The dots each represent one of the 81 model runs. The yellow star denotes the mean result of all 81 runs. The red diamond shows the main model result as presented in the main text. The median is shown by the vertical line in the box, and the box itself spans across the interquartile range from the first and third quartile. The whiskers extend to the smallest and largest values within 1.5 times the interquartile range. Source: Own analysis.

In comparison with the main model results (represented by the red diamond in both figures), the sensitivity analysis hardly yields any changes in results for regions outside of Europe or even globally (Supplementary Figure 5). However, the results for the European Union (representing Europe) exhibit much higher sensitivity of results. This is explored in more detail in Supplementary Figure 6 by holding one variation constant while still observing the variation across the three levels in the remaining parameters. Supplementary Figure 6 shows that model results for the EU are particularly sensitive to changes in the magnitude of yield dependency on pollination services and wild pollinator contribution to crop yields. The model parameters

have both lower influence on the model results. Especially, changes in the supply elasticity hardly change the results, while changes in the trade elasticity have a more marked effect. When assuming a lower degree of substitutability between foreign and domestic goods by reducing the Armington trade elasticity values, domestic production experiences less decline. However, this adjustment results in higher consumer prices, benefiting local producers but negatively impacting consumer surplus. Consequently, while the decline in consumer surplus becomes more pronounced, the overall welfare decline is moderated due to a compensating increase in producer surplus. Among the tested parameters, the dependence ratios and the contribution of wild pollinators to total pollination services exert comparably high influence on model outcomes.

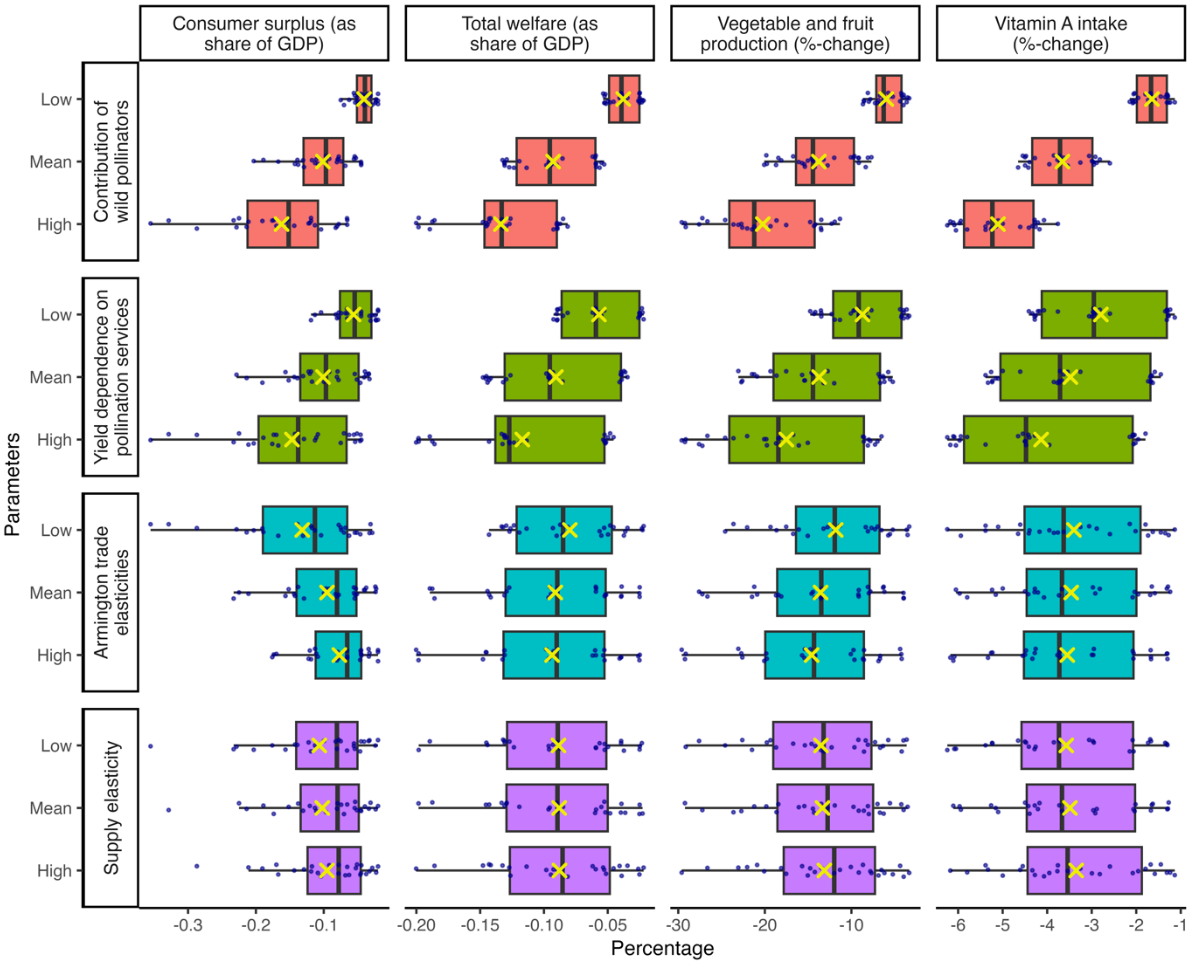

Supplementary Figure 6 - Sensitivity analysis of main model results in which the variation in one parameter is kept constant while the other three parameters are allowed to vary. The dots each represent one of the 81 model runs. The yellow star denotes the mean result of all 81 runs. The median is shown by the vertical box in the box, and the box itself spans across the interquartile range from the first and third quartile. The whiskers extend to the smallest and largest values within 1.5 times the interquartile range.

2. Calculation of managed pollinators replacement costs

Using data from FAOStat[10] and Chauzat et al[11], we estimate the number of colonies and honey production for each European country in 2017 (Supplementary Table 5). If a country did not report honey production, we assumed that its yield is equal the observed mean yield of 16.4 kg/colony. The production value of honey per country was estimated using the mean honeybee price of 5.20 EUR/kg, which resulted in a total value of honey production of 2.1 billion EUR for 2017. Alternatively, the cost of honey beekeeping in Europe is estimated based on the production cost per colony which is approximated by assuming it to be half of the cost reported for Germany (284 EUR/colony for a migratory beekeeping operation with 100 colonies). This results in a total production cost of honey beekeeping Europe of 3.6 billion EUR.

Supplementary Table 5 - Overview of bee colonies, honeybee production and production cost in Europe

Country	Estimated number of colonies	Estimated honey production (in metric tons)	Estimated production value at mean price of 5.20 EUR/kg (in million EUR)	Estimated production cost at 142 EUR/colony (in million EUR) [c]
Albania	289,962	3,614	18.8	41.2
Austria	353,300	5,800	30.2	50.2
Belarus	207,000	2,780	14.5	29.4
Belgium	33,314	545	2.8	4.7
Bosnia and Herzegovina	404,698	2,635	13.7	57.5
Bulgaria	754,105	11,807	61.4	107.1
Croatia	406,407	8,128	42.3	57.7
Czechia	637,553	9,365	48.7	90.5
Denmark[b]	91,736	1,500	7.8	13.0
Estonia	49,900	1,165	6.1	7.1
Finland	67,000	2,059	10.7	9.5
France	859,462	14,448	75.1	122.0
Germany	820,000	21,600	112.3	116.4
Greece	1,557,375	21,939	114.1	221.1
Hungary	816,000	32,000	166.4	115.9
Irelandb	16,371	268	1.4	2.3
Italy	1,473,843	9,500	49.4	209.3
Latvia	96,700	1,639	8.5	13.7
Lithuania	133,508	2,622	13.6	19.0
Luxembourg	5,266	93	0.5	0.7
Montenegro	65,000	390	2.0	9.2
Netherlands (Kingdom of the)[a,b]	70,000	1,145	6.0	9.9
North Macedonia	75,406	395	2.1	10.7
Norway[b]	76,447	1,250	6.5	10.9
Poland	1,571,299	17,089	88.9	223.1
Portugal	661,805	10,778	56.0	94.0
Republic of Moldova	148,080	4,000	20.8	21.0
Romania	1,437,394	30,177	156.9	204.1
Russian Federation	3,316,977	65,167	338.9	471.0
Serbia	849,280	7,014	36.5	120.6
Slovakia	278,385	4,212	21.9	39.5
Slovenia	86,500	804	4.2	12.3
Spain	2,904,971	29,393	152.8	412.5
Sweden	91,105	3,400	17.7	12.9
Switzerland	174,986	4,569	23.8	24.8
Ukraine[b]	4,050,528	66,231	344.4	575.2
United Kingdom[a]	200,000	8,977	46.7	28.4
**Total**	**25,131,663**	**408,496**	**2,124**	**3,569**

Note: Based on FAOstat data. [a] Colony data taken from Chauzat et al.[11], [b] Honey bee production imputed based on median yield of 16.4 kg/colony, [c] Based on the assumption that the average total production cost per colony is half of that reported for Germany[12].

3. Extrapolation of trends in hoverfly, flying insects or terrestrial insect populations in Europe

To discuss the plausibility of a wild pollinator collapse by 2030, we extrapolate available data on population trends on wild pollinators or broader taxonomic groups that include wild pollinators. To our knowledge, there is no generally agreed threshold that would define a "population collapse". In our context, we would argue that a wild pollinator collapse occurs if the population decline by more than 90% between 2017 and 2030 (14 years). This threshold is also used to assess the collapses in the biomass of fish stocks[13]. To extrapolate the trends, we assume an exponential decay function, which allows us to obtain annual change rates, γ, in the population based on a total change in that population, $\bar{\gamma}$, after T years.:

$$\gamma = (1 - \bar{\gamma})^{1/T} - 1$$

For $\bar{\gamma} = 90\%$ and $T = 14$, we obtain a threshold annual rate of decline of 15.2% according to which a population collapse would occur after 14 years.

The different recent studies reporting trends in pollinators of wild pollinator taxa or broader groups of insects that include wild pollinators are reported below (Supplementary Table 6). Studies on butterflies (*Lepidoptera)* were excluded, since they do not play a major role as pollinators[14].

Supplementary Table 6 - Overview of studies and their data on population trends

**Authors**	**Year study was published**	**Indicator**	**Location**	**Period**	**Years T**	**Total decline $\bar{\gamma}$,**	**Annual rate of change γ**
Barendgregt et al.[15]	2021	Hoverfly Abundance	NL	1982-2021	40	80%	-3.9%
Cooke et al.[16]	2023	Pollinator Occupancy	GB	1990-2018	29		-0.8%
Hallmann et al.[17]	2021	Hoverfly Abundance	DE	1989-2014	27	83%	-6.3%
Hallmann et al.[18]	2017	Flying Insect Biomass	DE	1989-2016	27	82%	-6.1%
Møller[19]	2019	Flying Insect Abundance	DK	1997-2017	22	89%	-9.5%
Powney et al.[20]	2019	Bee and Hoverfly Occupancy	GB	1980-2013	34	25%	-0.8%
Seibold et al.[21]	2019	Arthropod Biomass	DE	2008-2017	10	67%	-10.5%
Seibold et al.[21]	2019	Arthropod Abundance	DE	2008-2017	10	78%	-14.1%
van Klink et al[22,23]	2020	Terrestrial Insects	EUR	Since 2000	-	-	-2.3%
van Klink et al[22,23]	2020	Terrestrial Insects	EUR	Since 2005	-	-	-3.4%

The trends presented in Supplementary Table 6 are extrapolated in Supplementary Figure 7 below.

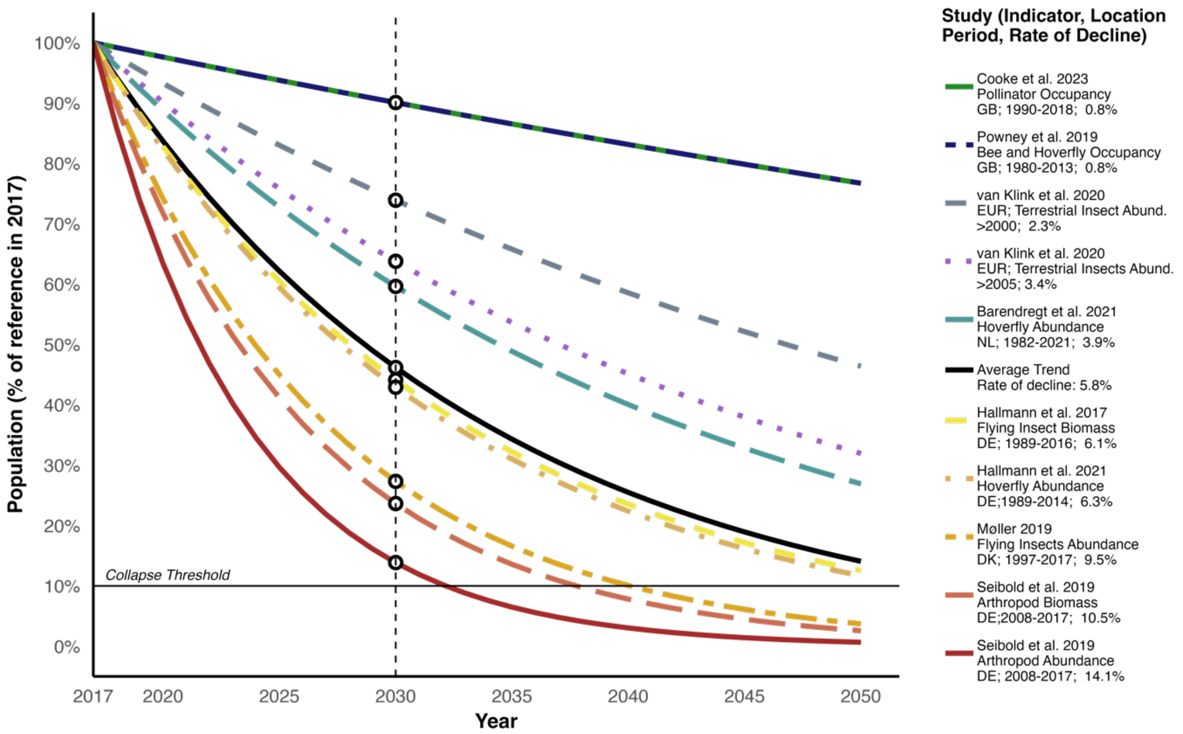

Supplementary Figure 7 - Extrapolated trends of hoverfly, flying insects or terrestrial insect populations in Europe from 2017 to 2030, derived using either observed change rates from studies or imputed change rates based on an exponential decay model. The grey horizontal line at 10% denotes the defined threshold of a collapse in wild pollinators.

References

1. Helming, J. F. M. & Terluin, I. J. *Scenarios for a Cap beyond 2013; Implications for EU27 Agriculture and the Cap Budget*. https://edepot.wur.nl/185179 (2011).
2. Overmars, K. P., Helming, J., Van Zeijts, H., Jansson, T. & Terluin, I. A modelling approach for the assessment of the effects of Common Agricultural Policy measures on farmland biodiversity in the EU27. *J. Environ. Manage.* **126**, 132–141 (2013) https://doi.org/ 10.1016/j.jenvman.2013.04.008.
3. CAPRI Consortium. CAPRI Model Documentation. (2022).
4. Siopa, C., Carvalheiro, L. G., Castro, H., Loureiro, J. & Castro, S. Animal-pollinated crops and cultivars—A quantitative assessment of pollinator dependence values and evaluation of methodological approaches. *J. Appl. Ecol.* **61**, 1279–1288 (2024) https://doi.org/ 10.1111/1365-2664.14634.
5. Klein, A.-M. *et al.* Importance of pollinators in changing landscapes for world crops. *Proc. R. Soc. B Biol. Sci.* **274**, 303–313 (2007) https://doi.org/ 10.1098/rspb.2006.3721.
6. European Parliament. *Minutes - Results of Roll-Call Votes - A9-0339/2023a*. https://www.europarl.europa.eu/doceo/document/PV-9-2023-11-22-RCV_EN.html (2023).
7. European Parliament. *Roll Coll Vote on the Proposal to Reject the Commission Proposal for a Regulation of the European Parliament and of the Council on Nature Restoration by Rapporteur César Luena*. https://www.europarl.europa.eu/doceo/document/PV-9-2023-07-12-RCV_EN.pdf (2023).
8. Jansson, T. *et al.* Coupled Agricultural Subsidies in the EU Undermine Climate Efforts. *Appl. Econ. Perspect. Policy* **43**, 1503–1519 (2021) https://doi.org/ 10.1002/aepp.13092.

9. Reilly, J. R. *et al.* Wild insects and honey bees are equally important to crop yields in a global analysis. *Glob. Ecol. Biogeogr.* **33**, e13843 (2024) https://doi.org/ 10.1111/geb.13843.
10. FAO. FAOstat database. (2024).
11. Chauzat, M.-P. *et al.* Demographics of the European Apicultural Industry. *PLoS ONE* **8**, e79018 (2013) https://doi.org/ 10.1371/journal.pone.0079018.
12. Kuratorium fuer Technik und Bauwesen in der Landwirtschaft, (KTBL). Wirtschaftlichkeitsrechner Tier - Honigbiene. (2024).
13. Edgar, G. J. *et al.* Stock assessment models overstate sustainability of the world's fisheries. *Science* **385**, 860–865 (2024) https://doi.org/ 10.1126/science.adl6282.
14. Rader, R. *et al.* Non-bee insects are important contributors to global crop pollination. *Proc. Natl. Acad. Sci.* **113**, 146–151 (2016) https://doi.org/ 10.1073/pnas.1517092112.
15. Barendregt, A., Zeegers, T., Van Steenis, W. & Jongejans, E. Forest hoverfly community collapse: Abundance and species richness drop over four decades. *Insect Conserv. Divers.* **15**, 510–521 (2022) https://doi.org/ 10.1111/icad.12577.
16. Cooke, R. *et al.* Protected areas support more species than unprotected areas in Great Britain, but lose them equally rapidly. *Biol. Conserv.* **278**, 109884 (2023) https://doi.org/ 10.1016/j.biocon.2022.109884.
17. Hallmann, C. A., Ssymank, A., Sorg, M., de Kroon, H. & Jongejans, E. Insect biomass decline scaled to species diversity: General patterns derived from a hoverfly community. *Proc. Natl. Acad. Sci.* **118**, e2002554117 (2021) https://doi.org/ 10.1073/pnas.2002554117.
18. Hallmann, C. A. *et al.* More than 75 percent decline over 27 years in total flying insect biomass in protected areas. *PLOS ONE* **12**, e0185809 (2017) https://doi.org/ 10.1371/journal.pone.0185809.

19. Møller, A. P. Parallel declines in abundance of insects and insectivorous birds in Denmark over 22 years. *Ecol. Evol.* **9**, 6581–6587 (2019) https://doi.org/ 10.1002/ece3.5236.

20. Powney, G. D. *et al.* Widespread losses of pollinating insects in Britain. *Nat. Commun.* **10**, 1018 (2019) https://doi.org/ 10.1038/s41467-019-08974-9.

21. Seibold, S. *et al.* Arthropod decline in grasslands and forests is associated with landscape-level drivers. *Nature* **574**, 671–674 (2019) https://doi.org/ 10.1038/s41586-019-1684-3.

22. van Klink, R. *et al.* Supplement to the Erratum by Van Klink et al. Preprint at https://doi.org/10.5281/ZENODO.4061806 (2020) https://doi.org/ 10.5281/ZENODO.4061806.

23. Van Klink, R. *et al.* Meta-analysis reveals declines in terrestrial but increases in freshwater insect abundances. *Science* **368**, 417–420 (2020) https://doi.org/ 10.1126/science.aax9931.
