# Supplementary material for: The economic, agricultural, and food security repercussions of a wild pollinator collapse in Europe
Source: Nat Commun. 2025 Nov 10;16:9892. doi: 10.1038/s41467-025-65414-7 (PMC12603210; doi:10.1038/s41467-025-65414-7)
Supplement: Supplementary file 2 — Description of Additional Supplementary Files [file 41467_2025_65414_MOESM2_ESM.pdf]

Description of Additional Supplementary Files

File Name: Supplementary Code 1
Description: Replication files consisting of the batch file, GAMS code and productivity shocks needed to replicate the scenarios within the CAPRI model.
