# Supplementary material for: The economic, agricultural, and food security repercussions of a wild pollinator collapse in Europe
Source: Nat Commun. 2025 Nov 10;16:9892. doi: 10.1038/s41467-025-65414-7 (PMC12603210; doi:10.1038/s41467-025-65414-7)
Supplement: Supplementary file 3 — Supplementary Code 1 [file 41467_2025_65414_MOESM3_ESM.zip › Readme.pdf]

README - Instructions for the Electronic Supplementary Materials of Feuerbacher et al. "The Economic, Agricultural, and Food Security Repercussions of a Wild Pollinator Collapse in Europe", accepted for publication in Nature Communications

A) Estimation of productivity shocks following a collapse of wild pollinators in Europe

1. Save the following three files (data_input.xlsx; Estimation of productivity shocks.gms; Estimation of productivity shocks_fromauthors.gdx) in one directory.
2. Open GAMS Studio (a free demo version can be obtained from https://www.gams.com/download/)
3. Open the file "Estimation of productivity shocks.gms";
4. Run the code
5. Inspect the shock parameters from the newly created gdx file
6. Optionally: Compare it with the values from the gdx file provided

B) Simulation of pollinator decline scenarios in the CAPRI model

The shock parameters are subsequently used for scenario analysis in the CAPRI model. The model can be downloaded from www.capri-model.org.

The CAPRI model comes with access to the underlying model database. The model database needs to be generated following the instructions of the CAPRI model website.

Once the reference model database has been estimated, the model can simulate the productivity shocks. The shocks for the collapse of wild pollinators in Europe are based on the dependence ratios from Siopa et al. (2024). They are provided in three different .gms files covering the mean productivity shock and the 95% confidence interval (either the low or the high dependence ratios for either end of the 95% CF interval):

1. Siopa_Europe_MeanDep_ContinentRegion.gms
2. Siopa_Europe_HighDep_ContinentRegion.gms
3. Siopa_Europe_LowDep_ContinentRegion.gms

These gms files are called from a batch file (Policy_Pollinators_siopa_wild_19_20Aug24.txt) within the graphical user interface (GUI) of the CAPRI model.

The results from these simulations can be viewed within the GUI. This will allow to analyze the main results. The results and graphics reported in the manuscript are further processed in the software package R.

The scenarios based on the dependence ratios taken from Klein et al. (2007) can be implemented in an analogous manner using the files provided.
